# A Dual Drug Sensitive *L. major* Induces Protection without Lesion in C57BL/6 Mice

**DOI:** 10.1371/journal.pntd.0002785

**Published:** 2014-05-29

**Authors:** Noushin Davoudi, Ali Khamesipour, Fereidoun Mahboudi, W. Robert McMaster

**Affiliations:** 1 Biotechnology Research Center, Dept of Medical Biotechnology, Pasteur Institute of Iran, Tehran, Iran; 2 Center for Research and Training in Skin Diseases and Leprosy, Tehran University of Medical Sciences, Tehran, Iran; 3 Department of Medical Genetics, University of British Columbia, Vancouver Coastal Health Research Institute, Vancouver, British Columbia, Canada; Queensland Institute of Medical Research, Australia

## Abstract

Leishmaniasis is a major health problem in some endemic areas and yet, no vaccine is available against any form of the disease. Historically, leishmanization (LZ) which is an inoculation of individual with live *Leishmania*, is the most effective control measure at least against cutaneous leishmaniasis (CL). Due to various reasons, LZ is not used today. Several live attenuated *Leishmania* have been developed but their use is limited. Previously, we developed a transgenic strain of *L. major* that harbors two suicide genes *tk* and *cd* genes (*lmtkcd^+/+^*) for use as a challenge strain in vaccine studies. These genes render the parasite susceptible to **Ganciclovir** (GCV) and 5-flurocytosine (5-FC). The dual drug sensitive strain of *L. major* was developed using gene targeting technology using a modified Herpes Simplex Virus thymidine kinase gene (*hsv-tk*) sensitive to Ganciclovir antibiotic and *Saccharomyces cerevisae* cytosine deaminase gene (*cd* sensitive to 5-flurocytosine) that were stably introduced into *L. major* chromosome. BALB/c mice inoculated with *lmtkcd^+/+^* developed lesions which upon treatment with GCV and 5-FC completely healed. In the current study, the transgenic *lmtkcd^+/+^*strain was assessed as a live vaccine model to determine the time necessary to develop a protective immune response. C57BL/6 mice were inoculated with the transgenic *lmtkcd^+/+^*strain, and treated at the time of inoculation (day0) or at day 8 after inoculation. Immunized animals were challenged with wild-type *L. major*, and complete protection was induced in mice that were treated at day 8. The results show that in contrast to leishmanization, in group of mice inoculated with a dual sensitive *L. major* development and persistence of lesion is not necessary to induce Th1 response and protection.

## Author Summary

Leishmaniasis is still a major health problem in some endemic foci, yet no vaccine is available against any form of leishmaniasis. It is a general belief that recovery from cutaneous leishmaniasis (CL) is accompanied with long life protection. An inoculation of live pathogenic *L. major* into healthy individuals to induce lesion similar to CL is called Leishmanization (LZ). Historically LZ showed to be the most effective control tool against CL. One of the drawbacks and reason for discontinuation of LZ was lesion development, which rarely lasts long. Treatment of CL is not an easy task. One line of development of an effective vaccine against leishmaniasis, a transgenic strain of *L. major* harboring two suicide genes *tk* and *cd* genes (*lmtkcd^+/+^*), was developed and previously checked in BALB/c mice. In this study, C57BL/6 mice were inoculated with transgenic *lmtkcd* ^+/+^strain; the rate of protection, parasite burden and the type of immune response were checked, and the results showed that complete protection induced by inoculation of *lmtkcd* ^+/+^strain if treatment is initiated on day 8 post inoculation.

## Introduction

Cutaneous leishmaniasis (CL) manifests as a localized self-healing lesion(s) that in rare cases develops to a non-healing lesion. If non-healing lesions develop, they are extremely difficult to treat with current therapies [1]. Control measures for leishmaniasis such as vector and/or réservoir control are not always practical, especially in remote endemic areas with limited resources. Efficacy of available drugs for leishmaniasis especially for CL is not acceptable and resistant is emerging [2], [3], [4], [5], [6]. Leishmanization (LZ) involves inoculating of individuals with live virulent *Leishmania major* to induce a single lesion that mimics a natural infection but with the lesion located at a predetermined site. Upon healing, the leishmanized individuals are protected against natural infection. LZ has been shown to be the most effective control measure at least against CL but the practice has been discontinued except on a limited scale in Uzbekistan. Primarily this is due to the development of chronic lesions that require medical intervention [7], [8], [9]. Despite ample evidence that development of an effective vaccine against leishmaniasis is possible there is still no vaccine available against any form of human leishmaniasis [10], [11], [12], [13]. One approach is to derive attenuated live vaccine strains of *Leishmania* through genetic manipulation to develop a parasite strain which has no virulence or a limited pathogenicity. A number of genetically manipulated *Leishmania* strains have been developed and studied in animal models with controversial results [14], [15], [16], [17], [18].

Previously, we developed a transgenic strain of *L. major* (*tk* ^+/+^ *–cd* ^+/+^)[*lmtkcd^+/+^*] harboring two suicide genes *tk* and *cd* genes that confer susceptibility to GCV and 5-FC,.as a challenge strain for vaccine studies. When BALB/c mice were inoculated in the flank with *lmtkcd^+/+^*, lesions developed at the site of inoculation, upon treatment with GCV and 5-FC complete healing occurred [16]. To extend these studies *lmtkcd^+/+^* was used to determine whether persistent infection is required for induction of a protective immune response against subsequent *L major* infection. The *lmtkcd^+/+^* promastigotes were inoculated into C57BL/6 mice and the inoculated mice were treated at set times with GCV to clear the infection. The mice were then challenged with wild type *L major*. Long term (3 months) complete protection against challenge with wild type *L major* was achieved with as little as 8 days vaccination time demonstrating that persistent infection is not required for complete protection.

## Materials and Methods

### Ethics statement

The ethical committee; Institutional Animal Care and Research Advisory Committee of Pasteur Institute of Iran, Education Office dated January, 2008, based on the Specific National Ethical Guidelines for Biomedical Research issued by the Research and Technology Deputy of Ministry of Health and Medicinal Education of Iran, issued in 2005, approved the protocol.

### Parasites

The *L. major* promastigotes (MHOM/IR/76/ER) used and from which the transgenic *lmtkcd^+/+^*parasites were derived, this *L. major* is the same isolate which was used for mass leishmanization, preparation of old world experimental vaccine and the *Leishmania* used for the skin test. Promastigotes were cultured in M199 medium (Life Technologies, Inc.) supplemented with 10% heat inactivated fetal calf serum (Gibco BRL) and 25 mM HEPES (Gibco BRL), pH 7 at 26°C. The parasite virulence was maintained by passage in BALB/c mouse.

### Mice

Female C57BL/6 mice, 6–8 week-old were purchased from the Animal Breeding Facility Centre (ABFC) of Pasteur Institute, Karaj, Iran. The animals were maintained in the animal facility of the Pasteur Institute of Tehran. The experiments were carried out according to the guidelines of Ethic Committee for Human use of Laboratory Animals, Pasteur Institute, Tehran, Iran.

### Infection, treatment and challenge

Mice were inoculated subcutaneously (SC) at the right hind footpad with 2×10^6^ stationary phase promastigotes of either *L. major* (MHOM/IR/76/ER) wild type (WT) or the transgenic *lmtkcd^+/+^*parasites in 50 µl PBS. The mice inoculated with *lmtkcd^+/+^*were divided into 3 groups and treated with a combination of GCV and 5Fcyt, 100 mg/Kg, intra-peritoneally (IP) either at the time of parasite inoculation (day 0), at day 8 after inoculation or for the control group which was left untreated. The dosage of the drugs used in this study was based on our previous study (17). The *lmtkcd^+/+^* inoculated groups were challenged in the left footpads with 2×10^6^ virulent WT *L. major* SC at 3 weeks after the end of the treatment period.

### Lesion development

The lesion development was recorded by weekly measurement of the footpad thickness at the site of inoculation using a metric caliper up to 12 weeks after inoculation.

### Parasite burden assay

Parasite burden was quantified once at week 10 after inoculation of the mice with either *L. major* wild type or with *lmtkcd^+/+^* and again 5 weeks after the challenge with wild type *L. major* (2–5 mice per group). The parasite burden in the spleen and draining lymph nodes were determined using limiting dilution analysis. To enhance sensitivity, 2-fold dilutions of the samples (up to 1/100) were used.

### DTH response

Delayed-type hypersensitivity (DTH) reaction was checked prior to challenge by injection of freeze-thawed (FT) *Leishmania major* (2×10^6^ promastigotes in 50 µl per injection) into the contralateral uninfected hind footpad. FT *L. major* promastigotes were prepared by repeating a freeze (−196°C)/thaw (37°C) cycle ten times. Footpad swelling was measured using a metric caliper at 24, 48 and 72 h after injection.

### Lymphocyte proliferation assay

Three mice from each group were sacrificed before and at 5 weeks after challenge inoculation, spleens were removed and cells cultured in complete RPMI-1640 medium in the presence or absence of 20 µg/well of Soluble *Leishmania* Antigens (SLA, 10^7^ *Leishmania* promastigotes/ml equal to 100 µg/ml) or Concavalin A (ConA;10 µg/ml) or without stimulation as a control.

### Cytokine assay

The levels of IFN-γ and IL-4 at weeks 5 and 10 post inoculation with *lmtkcd* ^+/+^ or WT *L.major* and 5 weeks after challenge were determined in the supernatant collected from spleen cell culture (5 mice per group). Briefly, single spleen cell suspension was prepared, cultured and re-stimulated either with SLA (100 µg/ml) or Con A (10 µg/ml). The supernatant was collected at 72 h. Then, the levels of IFN-γ and IL-4 were titrated using ELISA method according to the manufacturer's instruction (Bender Medsystems, Gmbh, Austria). The sensitivity of the ELISA kits was 3 pg/ml for IL-4 and 7.5 pg/ml for IFN- γ.

### Antibody response (IgG1 and IgG2a)

At week 5 after challenge, different groups of mice were tail bled and the levels of anti-*Leishmania* IgG1 and IgG2a Abs were checked by ELISA.

### Statistical analysis

All experiments were done in triplicates and the data was expressed as means ± S.E.M. The data was analyzed by one-way ANOVA followed by Tukey's test using SPSS V.13 software. *P* value<0.05 was considered as statistically significant.

## Results

### Footpad thickness after infection with WT *L. major* or *lmtkcd^+/+^*

**Figure 1:**
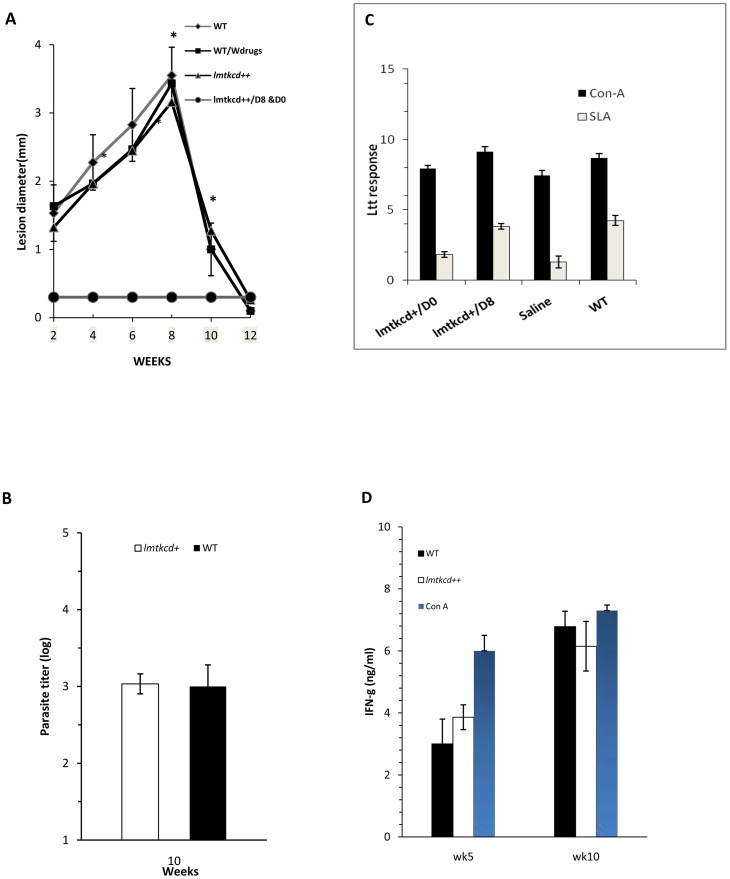
C57BL/6 mice were subcutaneously (SC) inoculated with either 2×10^6^ WT *L. major* or with *lmtkcd^+/+^* parasites and were treated with GCV/5-FCyt on day 0 (*lmtkcd^+/+^/D0*) or day 8 (*lmtkcd^+/+^/D8*) or left untreated. **A.** Lesion development was assessed by weekly measurement of footpad swelling. **B**. Parasite burden in groups of mice inoculated either with WT *L. major* or *lmtkcd^+/+^* was assessed at week 10. **C**. Lymphocyte transformation test was done on spleen cells. **D**. production of IFN-γ using ELISA method. Presented data are representative of 2 independent experiments.

C57BL/6 mice were inoculated SC with live wild type (WT) *L. major* parasites or *lmtkcd* **^+/+^** parasites and were either left untreated or treated with GCV/5-FCy at day 0 or day 8. Lesion development was followed by the measurement of footpad thickness. Following challenge with *L. major*, the protection rate and the immune responses generated were assessed. C57BL/6 mice inoculated with *lmtkcd^+/+^* or WT parasites and left untreated developed a similar lesion size which was cured around week 8–9. In contrast, no lesion was developed in the group of mice which was inoculated with *lmtkcd^+/+^* and received GCV/5-FCyt treatment at day 0 or day 8. The group of mice inoculated with WT *L. major* which was treated with GCV/5-FCyt developed a lesion similar to the untreated group of mice (Fig. 1A).

### Parasite burden after infection with WT *L. major* or *lmtkcd^+/+^*

**Figure 2:**
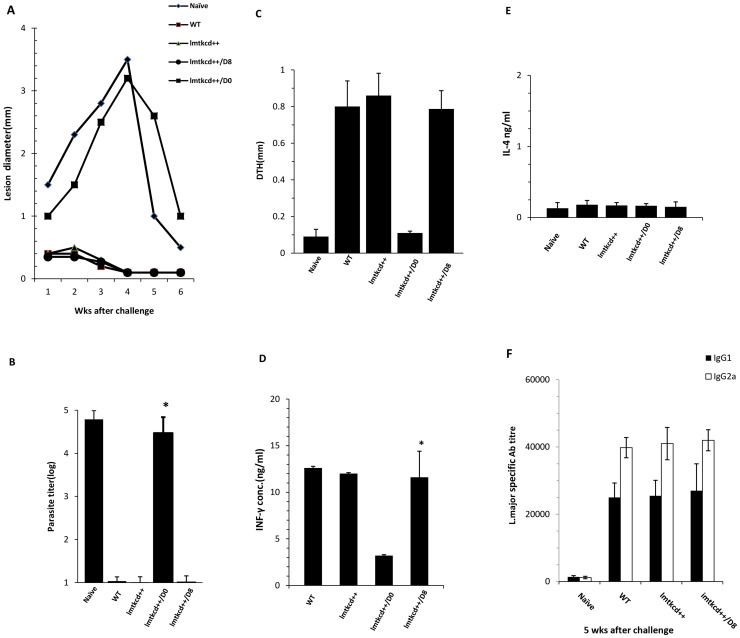
C57BL/6 mice with history of *L. major* infection or group of mice which were inoculated with *lmtkcd^+/+^ L. major* promastigotes and treated with GCV/5-Fcyt on day 0 or day 8 were subcutaneously (SC) challenged with 2×10^6^ wild type *L. major* along with a group of naive mice. **A**. Lesion development was assessed by weekly measurement of footpad swelling. **B**. Parasite burden was quantified in spleen on week 5 post challenge. **C.** DTH reaction was checked by measurement of footpad swelling at 72 hours after injection of freeze-thawed *L. major* into the contralateral uninfected hind footpad. Five weeks after challenge, the splenocytes were cultured and stimulated *in vitro* with SLA (100 µg/ml), Con A (10 µg/ml), or with no stimulation for 72 hrs. **D & E** The supernatants were collected and the levels of IFN-γ (D) and IL-4 (E) were titrated using ELISA, **F.** Anti *Leishmania* IgG1 and IgG2a at 5 weeks post challenge. Presented data are representative of 2 independent experiments.

The draining lymph nodes (LN) and spleen parasite burden was measured at week 10 post-inoculation (5 mice/group). The results showed no difference in the number of parasite in spleen and LN's in groups of mice inoculated with WT *L. major* and the group which was inoculated with *lmtkcd* ^+/+^ and received no treatment, the parasite burden of spleen at week 10 after inoculation is presented in Fig. 1B and only parasite burden of spleen at week 5 after challenge with WT *L. major* is presented in Fig. 2B.

### Immune response assay after infection with WT *L. major* or *lmtkcd^+/+^*

At weeks 5, 10 post-inoculation and week 5 post challenge mice (5 per group) were sacrificed and spleens were removed. A single cell suspension of spleen was prepared and cultured in the presence of either SLA (100 µg/ml), Con A (10 µg/ml) or without additional stimulation, lymphocyte transformation test (LTT) was done at 72 hours and the results showed a significantly (p<0.05) stronger LTT in group of mice with history of *L. major* infection and the group which was inoculated with *lmtkcd^+/+^*parasites and treated on day 8 than the group of mice inoculated with *lmtkcd^+/+^*parasites and treated on day 0 (Fig. 1C). The supernatants were collected and the levels of IFN-γ were titrated (Fig. 1D). Similar levels of IFN-γ were produced in spleen cells of group of mice inoculated with WT *L. major* and the group of mice inoculated with *lmtkcd^+/+^*. The level of IL-4 production was low and similar in group of mice inoculated with wild-type *L. major* or inoculated with *lmtkcd* ***^+/+^*** at week 16 post infection (data not shown).

### Challenge with WT *L. major*

To assess whether groups of C57BL/6 mice inoculated with *lmtkcd^+/+^* parasites are protected against WT *L. major* challenge, at week 5–6 post inoculation (3 weeks after the end of treatment upon commencing time), the groups of mice which received *lmtkcd^+/+^* and were treated on day 0 or 8 were challenged with *L. major*. As well, a group of mice which had healed spontaneously after *L. major* infection and a group of naïve mice were inoculated with *L. major* as controls. The results showed that the group of mice which was inoculated with *lmtkcd^+/+^* parasites and treated with GCV/5-Fcyt on day 8 and then challenged with WT at week 6, did not develop any lesion or swelling similar to the group of mice challenged with *L. major* after previously self-healing lesion. In contrast, the group of mice which was inoculated with *lmtkcd^+/+^* and treated at the same time (Day 0) with GCV/5-Fcyt and the group of naïve mice inoculated with *L. major* developed lesions (Fig. 2A).

### Parasite burden post-challenge with wild *L. major*

The parasite burden was quantified in draining LN at week 5 post-challenge with *L. major*, as shown in Fig. 2B. The number of parasites isolated from the group of mice which was inoculated with *lmtkcd^+/+^* and treated at day 8 with GCV/5-FCyt and the group of mice which had previously self-healed following *L. major* infection was significantly (p<0.05) lower than the group which was inoculated with *lmtkcd^+/+^* and treated at the same time (day 0) and the group of naïve mice which were inoculated with *L. major* for the first time. The number of parasites was very low in the groups of mice inoculated with either *lmtkcd^+/+^* and treated at day 8 or inoculated with *lmtkcd^+/+^* and not treated or the group of mice with history of *L. major* infection or the group of mice which were inoculated with *lmtkcd^+/+^* and treated at day 0, no significant difference was seen between the number of parasite in these groups.

### Immune response evaluation (DTH and cytokine assay)

DTH was done in different group of mice by injection of freeze-thawed (FT) *Leishmania major* (2×10^6^ promastigotes in 50 µl per injection) into the contra lateral uninfected hind footpad. The results are presented in Fig. 2C, a similar strong DTH response is seen in group of mice inoculated with WT *L. major*, or inoculated with *lmtkcd^+/+^* and treated with GCV/5-FCy on day 8 or left untreated, a low DTH response was seen in groups of mice inoculated with *lmtkcd^+/+^* and treated with GCV/5-FCy on day 0 or uninfected naïve mice. At week 10 after inoculation (before challenge) and 5 weeks after challenge, the splenocytes were cultured, stimulated *in vitro* with either SLA (100 µg/ml), or Con A (10^5^ µg/ml), or left unstimulated. LTT was done and the culture supernatants were collected at 72 hours and the level of IFN-γ and IL-4 was titrated using ELISA method. A significantly (p<0.05) stronger LTT was seen in mice with history of *L. major* infection and the group which was inoculated with *lmtkcd^+/+^* parasites and treated on day 8 than the group of mice inoculated with *lmtkcd^+/+^* parasites and treated on day 0 (data not shown). The level of IFN-γ was significantly higher in groups of mice inoculated with WT *L. major* or inoculated with *lmtkcd^+/+^* and treated with GCV/5-FCy on day 8 or left untreated (Fig. 2D). The level of IL-4 was similar in all the groups (Fig. 2E).

### IgG response

Serum samples were collected at 5 weeks after challenge, the results are presented in Fig. 2F, as shown a significantly (*P* = 0.002) higher anti-*L.major* IgG antibodies were seen in the group of mice with history of *L. major* lesion or group of mice inoculated with *lmtkcd^++^* and treated with GCV/5-FCy on day 8, in comparison with the group of naïve mice or group of mice inoculated with *lmtkcd^+/+^* and treated with GCV/5-FCy on day 0. IgG1 and IgG2a showed a significant (P = 0.001) increase after challenge compared to before challenge in all the groups and no significant difference was seen between the groups.

## Discussion

Cutaneous leishmaniasis manifests as a self-healing skin lesion(s) in exposed parts of the body, the healing process for lesions depends upon the *Leishmania* species involved and the host immune response. Usually healing takes up to 2 years, but CL might not be cured for several years with currently available treatments. Choices of therapeutic treatments for CL are limited and not always effective, often requiring multiple injections, introduce side effects and control measure tools are not always practical and successful [1], [2], [3], [6], [19], [20], [21]. It is well established that individuals with a history of CL are protected against development of further CL lesion. CL lesion(s) development is accompanied by the induction of strong immune response shown by *in vivo* and *in vitro* tests (9, 21). Despite many studies on leishmaniasis, immunological surrogate marker(s) of protection is not well defined in human leishmaniasis [9], [10], [22]. There is ample evidence to suggest that development of an effective vaccine against leishmaniasis is possible, but so far no vaccine is available against any form of leishmaniasis. The results of phase 3 clinical trials using crude *Leishmania* as vaccine were not promising [4], [12], [23], [24]. It has been shown that *in vitro* CD4^+^/CD8^+^ T-cell responses to live *Leishmania major* are significantly stronger than responses to dead parasites [25]. The only successful protective measure against CL has been shown to be leishmanization. One of the major drawbacks of LZ is the development of a lesion which might not heal during the expected time period and not respond to treatment [7], [9], [10]. Research have therefore focused on developing a *Leishmania* strain which upon inoculation does not induce a lesion or induces a lesion with limited pathogenicity, but at the same time maintains immunogenicity and as such induce protection in which the leishmanized individuals upon natural infection induce no lesion or even a limited fast healing lesion. In this regard attenuated and genetically manipulated *Leishmania* were developed and showed to induce protection in murine model of leishmaniasis [4], [15], [16], [26], [27]. Co-inoculation of *Leishmania* with CpG ODN showed to reduce the pathogenicity, but yet no *Leishmania* preparation reached to human use [28], [29].

Previously, the same group developed a recombinant double drug sensitive strain of *lmtkcd^+/+^* by integration of a genetically engineered HSV *tk* gene to confer sensitivity to GCV, and the *S. cerevisiae cd* gene to induce sensitivity to 5-fluorocytosine. Inoculation of BALB/c mice with *lmtkcd^+/+^* induces lesion similar to WT *L. major*, but the lesion was controllable by treatment with GCV/5-FCyt [16]. BALB/c mice does not mimic human CL so in the current study, C57BL/6 strain which is not a perfect model of human CL but more mimic the disease is used. Leishmanization which is an inoculation of virulent *L. major* in a predetermined part of the susceptible individuals, LZ induces a lesion similar to natural infection, protection against further multiple lesions is usually developed upon cure of the lesion caused by LZ and so far LZ showed to be the most effective preventive measure against CL. The main drawback of LZ is development of lesion [16].

Using drug sensitive *Leishmania* mimic natural infection similar to LZ and at the same time due to sensitivity of *Leishmania* to approved drugs assures a controllable lesion. As it is presented in Fig. 1, C57BL/6 mice inoculated with *L. major lmtkcd^+/+^* showed a lesion similar to WT *L. major* (Fig. 1A, Fig. 2A) with no difference in parasite burden (Fig. 1B, Fig. 2B). A very low number of *Leishmania* parasite is detected in the group of mice inoculated with *lmtkcd^+/+^* and treated with GCV/5-FCyt, A small number of *Leishmania* was detected in spleen of C57BL/6 mice long after recovery from *L. major* infection (unpublished data). A similar Th1 response was induced shown by LTT (Fig. 1C), DTH (Fig. 2C) and the cytokine levels of IFN-γ (Fig. 1D, Fig. 2D) and IL-4 (Fig. 2E) in groups of mice inoculated with WT and group of mice inoculated with *lmtkcd^+/+^*and treated with GCV and 5-FCyt on day 8 or left untreated, although in the group of mice inoculated with *lmtkcd^+/+^*and treated with GCV/5-FCyt on day 8, no lesion was developed at the site of inoculation but the reason for small increase in the size of footpad swelling is due to a slight inflammation which induced at the site of inoculation. Upon challenge with *L. major*, no lesion was developed and strong protection was seen similar to the group of mice cured from *L. major* infection (Fig. 2 A). The results showed that despite of no lesion development which was due to under control of recombinant *L. major* with ganciclovir and 5-Flourocytosin, strong Th1 immune response and protection against WT *L. major* was induced.
